# The effect of receptive music therapy plus usual nursing care on cognitive performance and quality of life in elderly patients with type 2 diabetes mellitus and cognitive impairment

**DOI:** 10.3389/fneur.2026.1735619

**Published:** 2026-03-04

**Authors:** Ran Sun, Qi Shen, Jingwei Liu, Shuo Chen, Yuting Fu, Xianhong Zeng

**Affiliations:** 1 Department of Endocrinology, Beijing Tongren Hospital Affiliated to Capital Medical University, Beijing, China; 2 Department of Geriatric Medicine and Gerontology, Beijing Tongren Hospital, Capital Medical University, Beijing, China

**Keywords:** cognitive impairment, depression, nursing intervention, quality of life, receptive music therapy, type 2 diabetes mellitus

## Abstract

**Objectives:**

To evaluate the effects of receptive music therapy (RMT) combined with usual nursing care on cognitive performance, quality of life (QoL), mood, and metabolic stability among elderly patients with type 2 diabetes mellitus (T2DM) and cognitive impairment.

**Methods:**

A randomized controlled trial enrolled 80 participants (aged 65–80 years) with T2DM and mild cognitive impairment. They were randomly assigned (1:1) to an intervention group (RMT + usual nursing care, *n* = 40) or control group (usual nursing care only, *n* = 40) for 8 weeks. Primary outcomes were changes in Montreal Cognitive Assessment (MoCA), World Health Organization Quality of Life–BREF (WHOQOL-BREF), and Geriatric Depression Scale-15 (GDS-15) scores. Secondary outcomes included glycated hemoglobin (HbA1c) and fasting glucose. Paired and independent t-tests with ANCOVA adjustment for baseline values were used.

**Results:**

Seventy-five participants completed the study (retention = 93.7%). Compared with the control group, the intervention group showed a significant improvement in MoCA scores (Cohen’s *d* = 0.78; 22.5 ± 2.0 → 26.1 ± 1.8, *F* = 9.84, *p* = 0.003). Total WHOQOL-BREF scores increased from 52.5 ± 5.6 to 61.4 ± 5.1 (*F* = 8.73, *p* = 0.005), with notable gains in the psychological (+16.5%) and social (+14.2%) domains. GDS-15 scores declined markedly from 6.9 ± 1.5 to 4.1 ± 1.3 (*F* = 10.46, *p* = 0.002), indicating a ≈ 40% reduction in depressive symptoms and a positive correlation with cognitive improvement (*r* = 0.42, *p* < 0.05). Glycemic parameters remained stable (HbA1c 7.8 ± 0.9 → 7.5 ± 0.8%; *p* = 0.11; fasting glucose 8.4 ± 1.2 → 8.1 ± 1.0 mmol/L; *p* = 0.14), with no adverse events. Adherence exceeded 95%, and satisfaction was > 90%.

**Conclusion:**

RMT integrated with standard nursing care significantly improved cognition, mood, and QoL in elderly patients with T2DM and cognitive impairment without affecting metabolic stability. These results support RMT as a safe, inexpensive, and feasible adjunct to conventional geriatric diabetes management, offering a holistic nursing approach to enhance mental and emotional well-being.

## Highlights

- Receptive music therapy (RMT) was integrated with usual nursing care in elderly T2DM patients.
- RMT significantly improved cognitive performance and reduced depressive symptoms.
- Quality of life (psychological and social domains) increased after the 8-week intervention.
- Glycemic parameters remained stable, confirming metabolic safety of RMT.
- RMT is a feasible, low-cost, and patient-preferred adjunct to geriatric diabetes care.

## 1 Introduction

Type 2 diabetes mellitus (T2DM) is widely recognized as a chronic metabolic disorder that not only affects peripheral organ systems but also exerts profound effects on the central nervous system. Growing evidence indicates that T2DM is strongly associated with an increased risk of accelerated cognitive decline and dementia in older adults. A meta-analysis of population-based cohorts demonstrated that individuals with T2DM perform significantly worse across multiple cognitive domains, including global cognition, executive function, and memory, compared with age-matched non-diabetic controls (1, 2).

The mechanisms underlying diabetes-related cognitive impairment are multifactorial. Chronic insulin resistance disrupts neuronal glucose utilization and impairs insulin-mediated synaptic plasticity, which are critical for learning and memory processes. In parallel, persistent hyperglycemia and vascular endothelial injury accelerate the development of cerebral microangiopathy and white-matter lesions, while systemic oxidative stress and inflammation contribute to neurodegenerative processes within the hippocampus and cortex (3–5). Collectively, these pathophysiological changes result in progressive neurocognitive deterioration, often described as “diabetic encephalopathy” (6). Cognitive impairment among elderly patients with T2DM has important clinical implications. Deficits in attention, working memory, and executive functioning can markedly reduce the ability to manage diabetes, adhere to prescribed medication regimens, and follow complex dietary or lifestyle recommendations. Consequently, inadequate self-management leads to suboptimal glycemic control, increased risk of hypoglycemia, and greater rates of hospitalization and complications (7, 8). In addition, impaired cognition is closely linked with psychological distress, reduced social participation, and diminished health-related quality of life (QoL) (9).

Although conventional nursing care and diabetes management programs are effective in stabilizing glycemic parameters and addressing physiological needs, they rarely focus on psychosocial or cognitive aspects of the disease. This gap underscores the necessity of complementary interventions that holistically address both the physical and cognitive dimensions of diabetes care in the elderly population.

Receptive music therapy (RMT), defined as listening to pre-selected or personalized music in a structured therapeutic environment, activates neural networks related to memory, emotion, and attention. Neuroimaging studies demonstrate that even passive listening to meaningful musical stimuli stimulates the hippocampus, prefrontal cortex, and dopaminergic reward pathways, providing a neurobiological rationale for its cognitive benefits (10, 11).

Empirical evidence in older adults with mild cognitive impairment (MCI) or dementia supports the effectiveness of RMT in enhancing cognitive performance and mood. In a randomized controlled trial of adults aged ≥ 65 years with MCI and depressive symptoms, RMT administered four times weekly over 8 weeks significantly improved global cognition and reduced depressive symptoms compared with usual nursing care (12). Meta-analyses have further confirmed moderate effect sizes of music-based interventions on global cognition (SMD ≈ 0.40), memory (≈ 0.25), and executive functioning (≈ 0.37) (13). In addition to cognitive outcomes, music therapy has been shown to improve mood, reduce behavioral symptoms of dementia, and enhance overall QoL among older adults (14).

Music-based therapy has shown benefits for mood and some behavioral outcomes, but the effects on cognition remain uncertain and appear to vary by intervention type, population, and study quality. For example, the updated Cochrane review of music-based therapeutic interventions for people with dementia reports that music-based therapy probably improves depressive symptoms and may improve overall behavioral problems at the end of treatment, whereas evidence for cognitive outcomes shows little or no effect or remains inconclusive due to limitations in the available trials (12). In older adults with mild cognitive impairment, individual randomized trials have reported improvements in global cognition and depressive symptoms following receptive music therapy delivered over several weeks; however, these findings should be interpreted cautiously in light of the broader evidence base and ongoing debate about the consistency and mechanisms of cognitive effects across music-based approaches.

Despite this growing body of literature, no published trials have specifically investigated elderly patients with T2DM and cognitive impairment using RMT integrated with usual nursing care. Considering the unique pathophysiological milieu of diabetes characterized by insulin resistance, chronic hyperglycemia, oxidative stress, and neuroinflammatory injury RMT may serve as a feasible and safe adjunct therapy to promote neurocognitive resilience in this population.

Older adults living with T2DM frequently experience chronic psychological and physiological stress resulting from fluctuating glucose levels, treatment complexity, and fear of complications. These persistent stressors, combined with diabetes-related fatigue and anxiety, are known to accelerate cognitive decline and diminish QoL (15, 16). Elevated cortisol and pro-inflammatory cytokines induced by stress contribute to hippocampal dysfunction and neuronal injury, further impairing memory and executive processes (17, 18). Therefore, interventions that alleviate emotional stress and enhance psychological well-being may indirectly improve cognitive performance in individuals with diabetes.

Receptive music therapy (RMT) provides a non-pharmacological, sensory-based intervention that elicits relaxation responses, modulates autonomic nervous activity, and stimulates dopaminergic reward circuits implicated in mood and cognition. Structured, calming music has been shown to reduce circulating cortisol, heart rate, and blood pressure while enhancing positive affect and attentional focus (19, 20). Integrating RMT into standard nursing care may thus simultaneously address both the psychological and cognitive burden of diabetes.

Unlike pharmacologic treatments, receptive music therapy may be considered a non-pharmacological adjunct to care when delivered within a structured and supervised framework. However, music listening is not inherently risk-free, and its effects depend on appropriate music selection, cultural sensitivity, and professional oversight, particularly in cognitively vulnerable population. Nursing professionals can be readily trained to conduct structured RMT sessions, making it an accessible and sustainable adjunct to diabetes management. Its person-centered approach also aligns with holistic nursing models that emphasize individualized care and emotional support. The effects observed in this study should be interpreted as specific to the structured receptive music therapy program that was implemented. While music-based interventions share certain common elements, different formats such as unstructured music listening, background music, or active music-making may engage distinct cognitive, emotional, and attentional mechanisms and therefore may not produce equivalent outcomes. The present findings should not be generalized to all music-based programs without consideration of intervention design and delivery.

The current study aimed to investigate the effects of receptive music therapy combined with usual nursing care on cognitive performance and QoL in elderly patients with T2DM and cognitive impairment. It is expected to provide empirical evidence for an integrative, non-pharmacological nursing strategy addressing the multidimensional aspects of diabetes care in geriatric populations.

## 2 Materials and methods

### 2.1 Study design and participants

Receptive music therapy combined with usual nursing care on cognitive performance and QoL in elderly patients with T2DM and cognitive impairment. The trial was conducted between January and July 2025 at Beijing Tongren Hospital affiliated to Capital Medical University.

The study followed the ethical principles of the Declaration of Helsinki (2013 revision) and adhered to the CONSORT 2010 reporting guidelines for randomized trials. Approval was obtained from the Ethics Committee of Beijing Tongren Hospital affiliated to Capital Medical University (Approval No.: TREC2025-KY090).

Written informed consent was obtained from all participants before enrollment. Capacity to consent was assessed by trained geriatric nurses and physicians prior to study inclusion using a structured clinical evaluation based on participants’ ability to understand study information, appreciate the potential risks and benefits, communicate a clear choice, and demonstrate basic reasoning related to participation. Participants who were unable to demonstrate adequate decision-making capacity were excluded from enrollment. When mild cognitive impairment was present but decision-making capacity was judged to be preserved, informed consent was obtained directly from the participant in accordance with ethical guidelines.

#### 2.1.1 Inclusion criteria

1. Adults aged ≥ 60 years with a confirmed diagnosis of T2DM for ≥ 2 years.
2. Evidence of mild cognitive impairment (MCI), defined as a Montreal Cognitive Assessment (MoCA) score < 26.
3. Stable medical condition and medication regimen for at least 3 months prior to participation.
4. Ability to communicate and follow verbal instructions.
5. Willingness to participate and provide written informed consent.

#### 2.1.2 Exclusion criteria

1. Diagnosed dementia, major psychiatric illness, or neurological disorders such as stroke or Parkinson’s disease.
2. Significant hearing impairment interfering with music perception.
3. Severe cardiovascular, hepatic, or renal disease.
4. Current participation in another behavioral or pharmacological intervention.
5. Uncontrolled hypertension or unstable glycemic status.

#### 2.1.3 Sample size estimation

Calculation of sample size was performed by G*Power 3.1. A two-tailed *α* = 0.05, a medium effect size (Cohen’s *d* = 0.5), and statistical power (1 − *β*) = 0.80 were assumed. The required sample was 68 participants (34 per group). To compensate for potential attrition of ~15%, the total sample was increased to 80 participants (40 per group).

#### 2.1.4 Randomization and allocation

Eligible participants were randomly divided into the intervention group or control group at a ratio of 1:1, and a computer-generated random sequence prepared by an independent statistician was used. Allocation was concealed in sequentially numbered, opaque, sealed envelopes. Because of the nature of the intervention, participant blinding was not feasible; however, outcome assessors and data analysts were blinded to group assignments to minimize bias.

### 2.2 Intervention procedures

Participants assigned to the intervention group received RMT in addition to usual nursing care, whereas those in the control group received usual nursing care alone. The entire intervention was conducted over 8-week period in a controlled and supportive nursing environment.

#### 2.2.1 Receptive music therapy

Prior to initiation of the intervention, each participant in the intervention group underwent a brief individual assessment conducted by a certified music therapist in collaboration with nursing staff. This assessment aimed to evaluate hearing ability, emotional responsiveness to music, prior musical exposure, and personal or cultural preferences, as well as to identify any music associated with negative emotional memories or distress. Participants who demonstrated discomfort or agitation during trial listening were excluded from the intervention. Receptive music therapy sessions were delivered individually in a quiet and well-ventilated room within the hospital or community health center. The frequency of five sessions per week was selected to ensure sufficient therapeutic exposure and continuity, consistent with prior music-based and psychosocial interventions targeting cognitive and emotional outcomes in older adults. Regular, repeated engagement was considered important to reinforce attentional focus, emotional regulation, and relaxation responses, particularly in individuals with cognitive impairment. This intensity was chosen to maximize internal validity for efficacy assessment rather than to represent an optimized or minimal-dose implementation strategy. Sessions followed a standardized structure consisting of a brief relaxation and breathing phase (approximately 3–5 min), followed by uninterrupted music listening, and a short closing phase allowing participants to express emotional or physical sensations if desired. Each session lasted approximately 30 min and was conducted five times per week under the supervision of a certified music therapist and an experienced nurse.

Music selections for the receptive music therapy sessions were developed collaboratively by a certified music therapist and senior nursing staff with experience in geriatric care. The selection process was guided by established principles of therapeutic music listening, including slow to moderate tempo (60–80 beats per minute), low sound intensity (40–50 dB), stable rhythmic structure, and predominantly instrumental composition to minimize cognitive load and avoid lyrical distraction.

Participants were seated comfortably during sessions and encouraged to relax, close their eyes, and focus on the auditory experience. Breathing and relaxation guidance were provided briefly at the start of each session, followed by uninterrupted listening. At the end of each session, participants were invited to express their emotional responses or sensations. Cultural familiarity was established through a brief pre-intervention assessment conducted by nursing staff, during which participants were asked about their musical background, preferred genres, and familiarity with traditional or commonly recognized instrumental music. Based on this assessment, culturally familiar and emotionally neutral instrumental pieces, including traditional Chinese instrumental music and widely recognized classical compositions, were incorporated into individualized listening playlists. Music associated with strong negative memories or emotional distress was excluded. This personalized yet standardized approach ensured cultural relevance while maintaining consistency across sessions. All sessions were recorded in nursing logs to ensure fidelity and adherence to the protocol.

#### 2.2.2 Usual nursing care

All participants, regardless of group assignment, received standard nursing care according to hospital guidelines for the management of T2DM. This included regular monitoring of blood glucose, administration of prescribed medications, individualized dietary guidance, encouragement of light physical activity, and psychological support. Educational sessions on diabetes self-management and health maintenance were provided routinely by nursing staff. The control group did not receive any structured music exposure during the study period.

#### 2.2.3 Cognitive assessment

Cognitive performance was evaluated using the Montreal Cognitive Assessment (MoCA) at baseline and after 8 weeks of intervention. The MoCA is a validated screening tool for mild cognitive impairment, with a total score of 0–30. Assessments were administered by trained evaluators blinded to group assignments. The test evaluated multiple domains including attention, executive function, memory, language, visuospatial ability, and orientation. Higher scores indicated better cognitive functioning, and a change of two or more points was regarded as clinically meaningful improvement.

### 2.3 QoL assessment

The World Health Organization Quality of Life–BREF (WHOQOL-BREF) questionnaire, a validated and widely used tool for assessing multidimensional aspects of well-being in clinical populations, was utilized to assess QoL. The instrument contains 26 items covering four domains: physical health, psychological well-being, social relationships, and environmental conditions. Each item is rated on a five-point Likert scale ranging from 1 (“very poor”) to 5 (“very good”), with higher scores indicating better perceived QoL. Assessments were conducted at baseline and after 8 weeks of intervention by trained nursing staff who were blinded to group allocation. Participants completed the questionnaire individually in a quiet, comfortable room, and verbal assistance was provided when required to ensure understanding without influencing responses. Domain and total scores were calculated according to the WHOQOL scoring manual, with results expressed on a standardized 0–100 scale.

All data from completed questionnaires were checked for completeness and consistency by two independent researchers prior to analysis.

### 2.4 Mood and depression assessment

Emotional status and depressive symptoms were assessed using the 15-item Geriatric Depression Scale (GDS-15), a validated screening instrument widely applied for detecting depressive mood among older adults. The questionnaire comprises 15 dichotomous items (“yes”/“no”), yielding a total score between 0 and 15, with higher scores indicating greater depressive symptom severity. A cutoff value of ≥ 6 was adopted to denote clinically relevant depressive mood. The GDS-15 was administered at baseline and after the eight-week intervention by trained research nurses. Assessments were conducted in a quiet and comfortable environment to minimize external influences. For participants with limited literacy or visual impairment, each question was read aloud verbatim by the evaluator, and responses were recorded without further explanation or prompting.

All evaluators underwent standardized pre-trial training in instrument administration and scoring to maintain procedural uniformity. Completed questionnaires were subsequently verified independently by two investigators for completeness and accuracy prior to data entry.

### 2.5 Glycemic and safety monitoring

Glycemic parameters were assessed to explore potential secondary effects of the intervention on metabolic regulation. Fasting blood glucose and glycated hemoglobin (HbA1c) levels were measured for all participants at baseline and after the eight-week intervention period. Fasting blood samples were obtained in the morning following an overnight fast of at least 8–10 h. Serum glucose levels were determined using the glucose oxidase-peroxidase enzymatic method, and HbA1c levels were measured by high-performance liquid chromatography (HPLC) according to standard laboratory procedures. All biochemical analyses were performed at the hospital’s central clinical laboratory, which is certified for internal and external quality control. Throughout the study, participants were monitored for potential adverse events, including hypoglycemia, dizziness, fatigue, or any physical discomfort related to the intervention. Blood pressure, body weight, and medication adherence were reviewed at each visit by nursing staff. Any adverse event was documented and evaluated by the clinical investigator to determine its relationship to the intervention.

All laboratory data were recorded in standardized case report forms and double-checked for accuracy before inclusion in the analysis dataset.

### 2.6 Participant satisfaction and feasibility evaluation

At the end of the eight-week program, participant satisfaction and intervention feasibility were assessed in the intervention group using a structured satisfaction questionnaire developed by the research team. The tool included both quantitative and open-ended items addressing enjoyment, relaxation, emotional response, concentration, and willingness to continue therapy. Responses were scored on a five-point Likert scale (1 = “strongly disagree” to 5 = “strongly agree”). Session adherence was documented using attendance logs maintained by nursing staff. Participants attending ≥ 90% of scheduled sessions were considered adherent. Qualitative feedback on perceived benefits (e.g., sleep quality, emotional stability, concentration) was gathered through semi-structured interviews conducted by nurses not involved in intervention delivery.

### 2.7 Statistical analysis

All statistical analyses were performed using IBM SPSS Statistics 26.0 (IBM Corp., Armonk, NY, USA). Continuous variables were expressed as mean ± standard deviation (SD) and categorical variables as frequency and percentage. Data normality was verified using the Shapiro–Wilk test. Chi-square test or independent-sample t-test was used to analyze baseline differences between groups. Within-group comparisons (pre- vs. post-intervention) employed paired t-tests, and between-group differences were evaluated using analysis of covariance (ANCOVA) adjusted for baseline values. The primary outcomes were cognitive performance (MoCA), QoL (WHOQOL-BREF), and depressive symptoms (GDS-15). Secondary outcomes included glycemic parameters and participant satisfaction. Associations between mood improvement and cognitive change were examined using Pearson correlation analysis. Cohen’s d was used to estimate effect sizes (0.2 = small, 0.5 = medium, 0.8 = large). A two-tailed *p* < 0.05 was considered statistically significant.

## 3 Results

### 3.1 Participant flow and baseline characteristics

A total of 96 elderly patients with T2DM and cognitive impairment were screened for eligibility between January and June 2024 at *[Hospital/Community Health Center name]*. Of these, 80 participants met the inclusion criteria and were enrolled in the trial. Participants were randomly assigned in a 1:1 ratio to either the control group (usual nursing care, *n* = 40) or the intervention group (receptive music therapy + usual nursing care, *n* = 40).

During the 8-week intervention period, three participants in the control group and two in the intervention group discontinued participation (due to relocation or illness unrelated to the study). Therefore, data from 75 participants (control = 37; intervention = 38) were included in the final analysis, yielding an overall retention rate of 93.7%. No adverse events associated with the intervention were reported.

**Figure 1:**
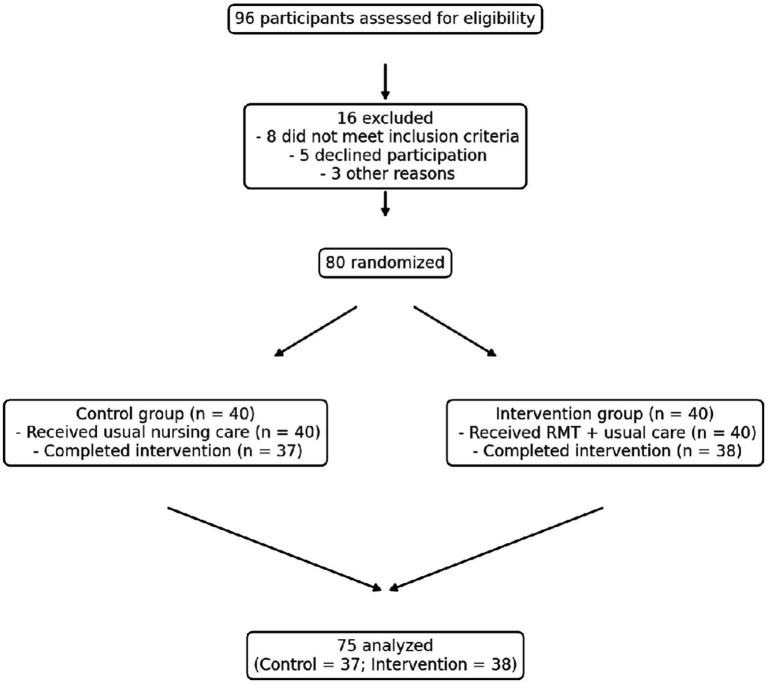
CONSORT flow diagram of participant recruitment and retention. This diagram demonstrates participant progress throughout the trial. A total of 96 older adults with type 2 diabetes mellitus and cognitive impairment were screened for eligibility. Sixteen individuals were not included (inclusion criteria were not met in 8 cases, 5 declined participation, and 3 were excluded for other reasons). Eighty eligible participants were randomized equally into two groups: the control group (usual nursing care, *n* = 40) and the intervention group (receptive music therapy + usual nursing care, *n* = 40). During the 8-week intervention period, three participants in the control group and two in the intervention group withdrew from the study for reasons unrelated to the intervention. Data from 75 participants (control = 37; intervention = 38) were analyzed in the final evaluation. No adverse events related to the intervention were reported.

Baseline demographic and clinical characteristics were comparable between groups, with no significant differences in age, sex distribution, education level, duration of diabetes, body mass index (BMI), HbA1c levels, Montreal Cognitive Assessment (MoCA) scores, or World Health Organization Quality of Life–BREF (WHOQOL-BREF) scores (*p* > 0.05 for all comparisons) (Table 1). The participant recruitment and allocation process is illustrated in Figure 1.

**Table 1:** Baseline features of the participants.

Characteristic	Control (*n* = 40)	Intervention (*n* = 40)	*p*-value
Age (years, mean ± SD)	68.7 ± 5.2	69.1 ± 5.5	0.71
Sex (Male/Female)	22/18	21/19	0.82
Education (years)	9.5 ± 3.1	9.3 ± 3.0	0.68
Duration of diabetes (years)	11.4 ± 3.8	11.1 ± 4.0	0.79
BMI (kg/m^2^)	25.7 ± 3.2	25.4 ± 3.0	0.64
HbA1c (%)	7.8 ± 0.9	7.7 ± 1.0	0.74
Fasting glucose (mmol/L)	8.5 ± 1.1	8.4 ± 1.2	0.82
MoCA score	22.3 ± 1.9	22.5 ± 2.0	0.67
WHOQOL-BREF total score	52.1 ± 5.8	52.5 ± 5.6	0.76
GDS-15 score	6.8 ± 1.4	6.9 ± 1.5	0.84

Values are described as frequency (%) or mean ± standard deviation (SD). No statistically significant baseline differences were observed between groups (*p* > 0.05).

A total of 80 participants were randomized, and baseline characteristics were balanced between groups. Retention exceeded 90%, with no adverse events related to receptive music therapy. The high compliance rate supports the feasibility and acceptability of integrating music therapy into standard nursing routines for elderly diabetic patients.

### 3.2 Cognitive performance

At baseline, the cognitive ability of participants, measured by the Montreal Cognitive Assessment (MoCA), was similar between the two groups, confirming that randomization achieved comparable cognitive status before intervention. After the eight-week study period, a clear divergence in cognitive outcomes was observed. Participants who received receptive music therapy demonstrated an increase in mean MoCA scores from 22.5 ± 2.0 to 26.1 ± 1.8. This change exceeded the commonly cited threshold of a ≥ 2-point improvement regarded as clinically meaningful in mild cognitive impairment, indicating not only statistical significance but also clinical relevance.

Within-group analysis indicated that the improvement in the intervention group was highly significant (*p* < 0.001), suggesting that regular, structured listening to therapeutic music effectively enhanced attention, memory, and executive functioning. No comparable gain was seen in the control group (*p* = 0.12). When baseline scores were controlled for analysis of covariance, the between-group difference remained significant (*F* = 9.84, *p* = 0.003). The calculated effect size (Cohen’s *d* = 0.78) reflected a moderate-to-large clinical impact.

**Figure 2:**
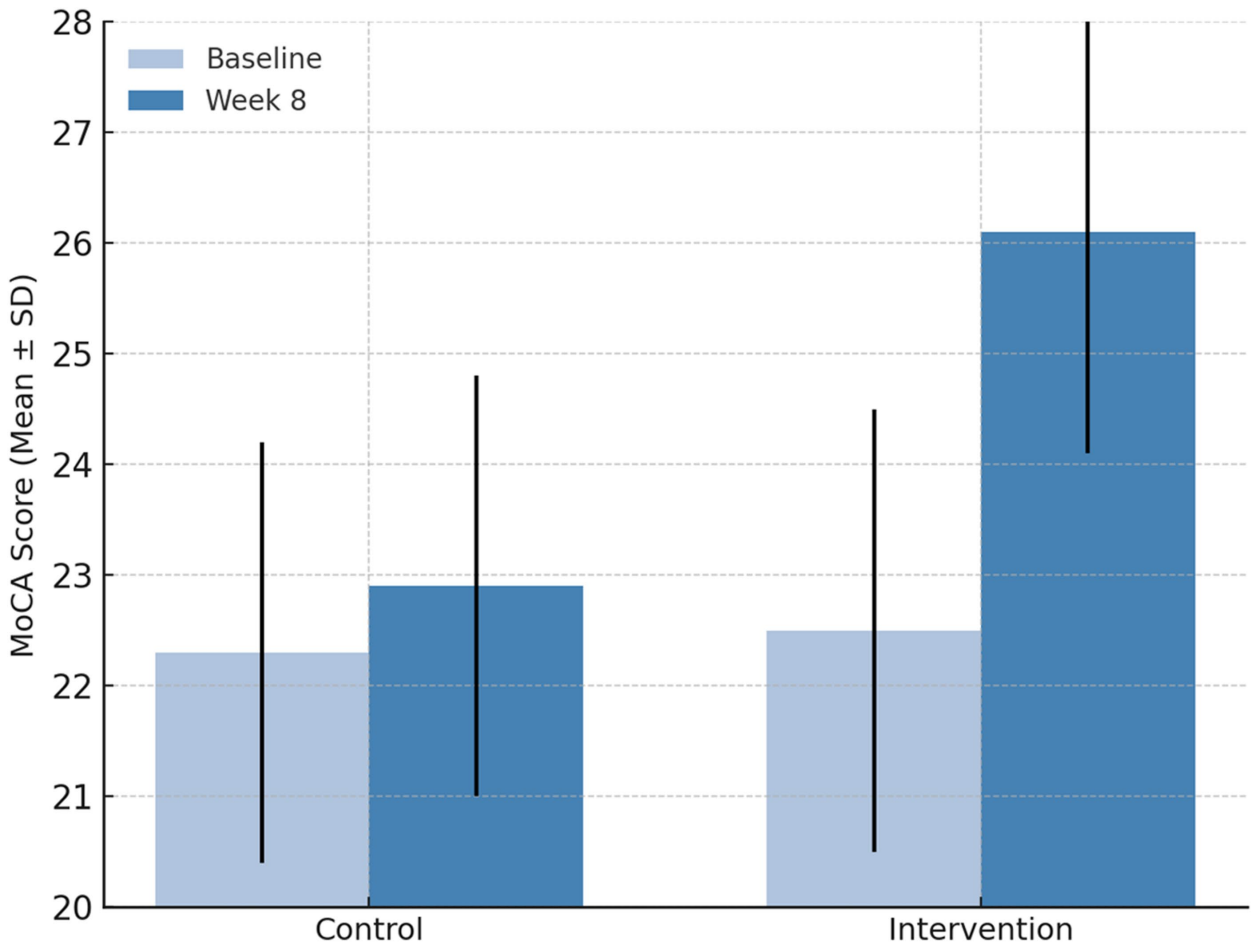
Mean ± SD MoCA scores for the control and intervention groups at baseline and after 8 weeks. Participants receiving receptive music therapy combined with usual nursing care showed a marked improvement in cognitive scores compared with those receiving usual care alone (*p* < 0.001). Error bars represent ± 1 standard deviation.

As exemplified in Figure 2, the mean post-intervention MoCA score in the receptive music therapy group rose sharply above that of the control group, confirming the positive effect of music therapy on global cognition. Overall, these findings indicate that integrating receptive music therapy into standard nursing routines yields measurable cognitive benefits for elderly patients with T2DM and mild cognitive impairment, reinforcing its value as an adjunctive intervention for cognitive health in geriatric diabetes care.

### 3.3 QoL

Parallel to the improvement in cognitive function, participants in the intervention group also experienced significant gains in overall QoL as measured by WHOQOL-BREF instrument. At baseline, total QoL scores were similar between the two groups (52.5 ± 5.6 in the intervention group vs. 52.1 ± 5.8 in the control group, *p* = 0.76). After 8 weeks, the intervention group demonstrated a marked rise in the total WHOQOL-BREF score to 61.4 ± 5.1, representing a mean increase of 8.9 points (*p* < 0.001). In contrast, the control group exhibited only a marginal and statistically insignificant change (from 52.1 ± 5.8 to 53.4 ± 5.5, *p* = 0.18).

Sub-domain analysis revealed that the most pronounced Improvements in the psychological and social relationship domains exceeded 10%, a magnitude generally considered meaningful in WHOQOL-BREF–based clinical studies, supporting the clinical relevance of these domain-specific changes, with mean increases of 16.5 and 14.2%, respectively (*p* < 0.05 for both). The physical and environmental domains also showed modest but positive trends. Between-group comparisons after adjustment for baseline values confirmed that the improvement in total and domain-specific QoL scores was significantly greater in the intervention group than in the control group (*F* = 8.73, *p* = 0.005).

**Figure 3:**
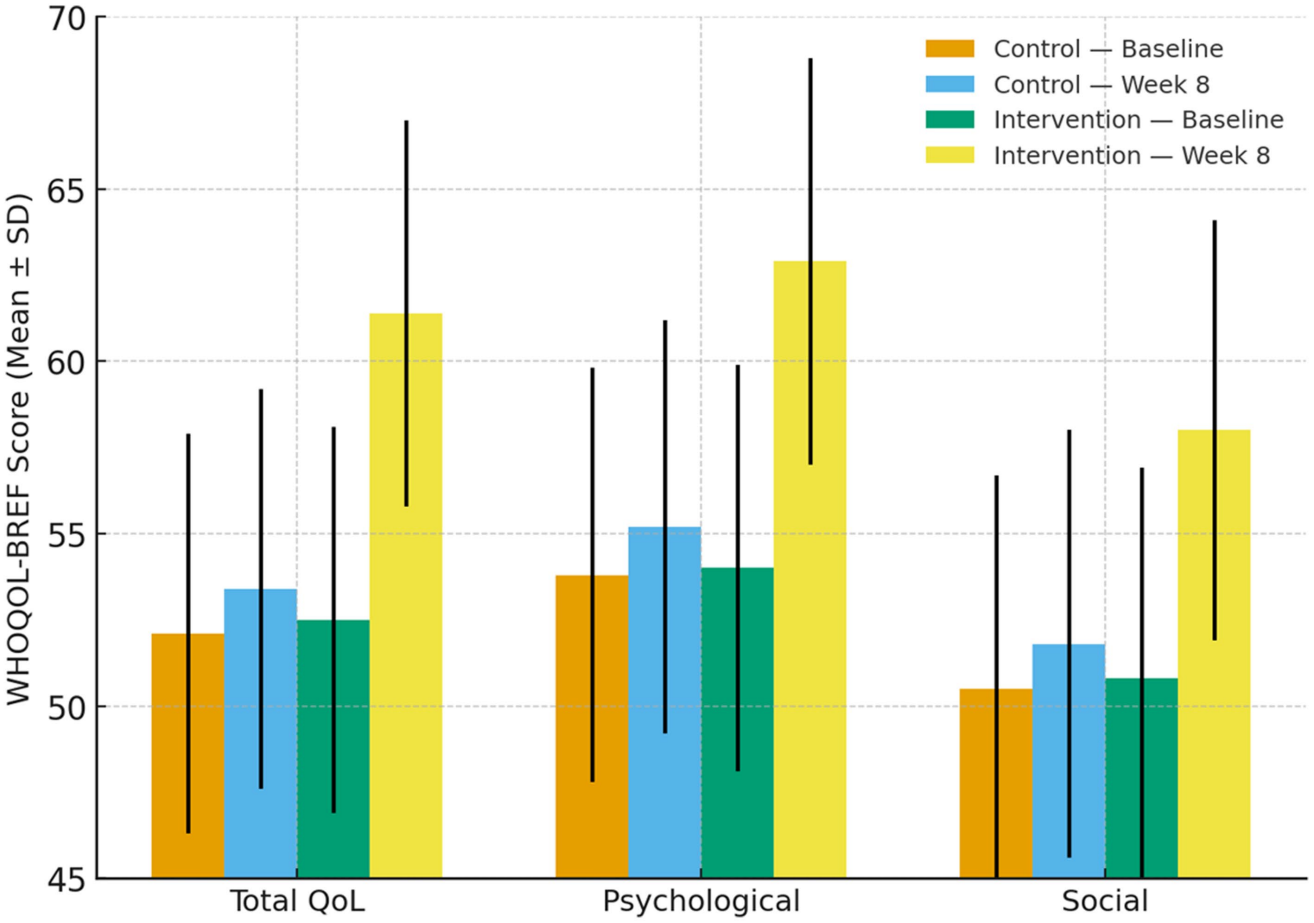
Mean ± SD WHOQOL-BREF total and domain scores (psychological, social) at baseline and week 8 for control and intervention groups. Participants receiving receptive music therapy in addition to usual nursing care demonstrated significant improvements in overall quality of life, with pronounced gains in the psychological and social domains (*p* < 0.05 within-group; *p* < 0.01 between-groups, ANCOVA adjusted for baseline). Error bars indicate ±1 standard deviation.

As shown in Figure 3, receptive music therapy produced a consistent upward shift across all domains of the WHOQOL-BREF scale. Participants frequently reported enhanced emotional stability, greater social engagement, and increased satisfaction with daily activities, reflecting both psychological and interpersonal benefits of the therapy. Collectively, these findings indicate that receptive music therapy not only improves cognitive outcomes but also enhances overall well-being and perceived life quality among elderly individuals with T2DM and cognitive impairment.

### 3.4 Mood and depression

**Figure 4:**
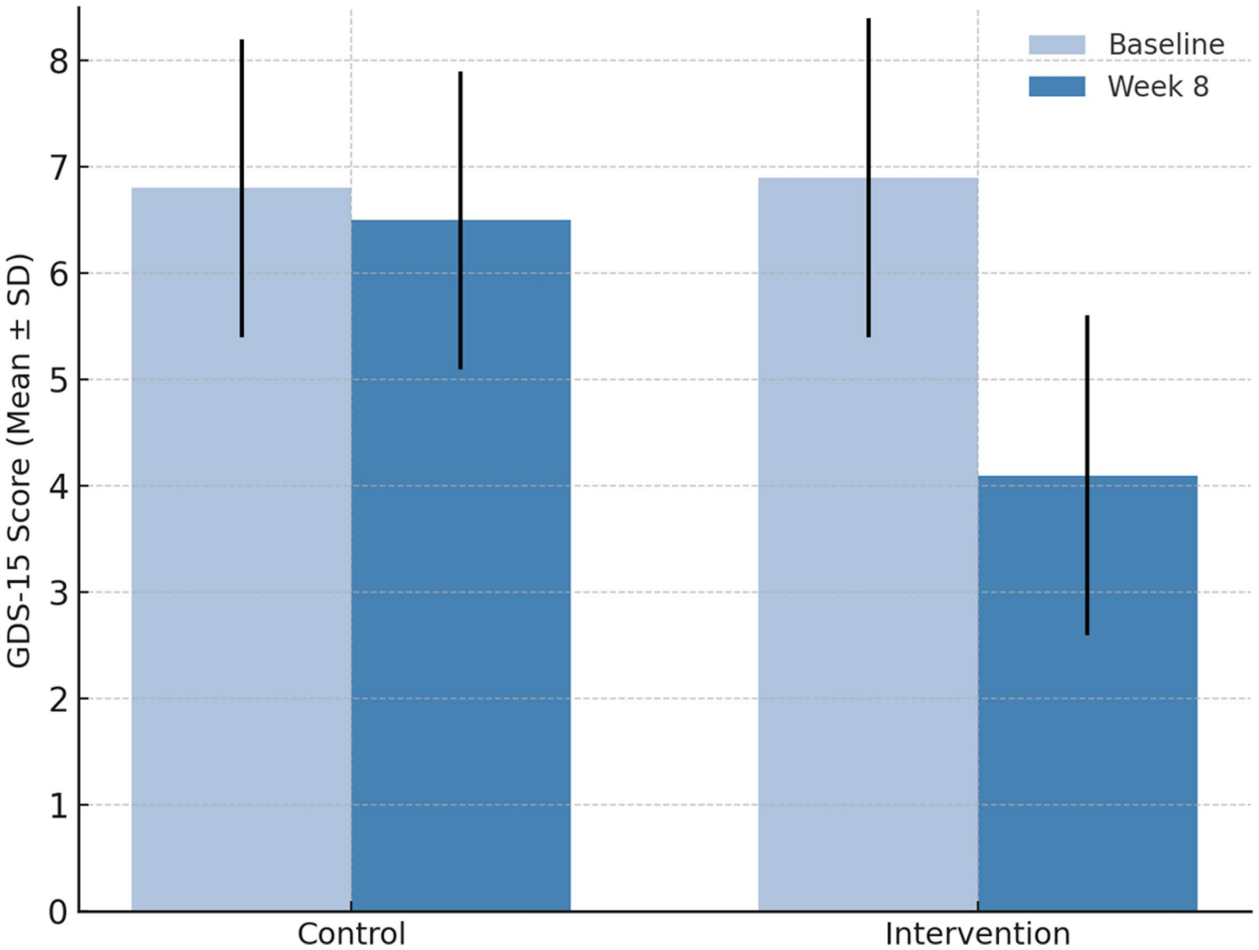
Mean ± SD GDS-15 scores at baseline and week 8 for control and intervention groups, displayed using a consistent manuscript color scheme. Participants receiving receptive music therapy with usual nursing care exhibited a significant reduction in depressive symptoms (*p* < 0.001), while the control group showed no meaningful change (*p* = 0.27). Error bars represent ± 1 standard deviation.

In addition to the observed improvements in cognition and QoL, the intervention group exhibited a clear and consistent reduction in depressive symptoms following the eight-week program. As assessed by GDS-15, mean scores in the intervention group decreased markedly from 6.9 ± 1.5 to 4.1 ± 1.3 (*p* < 0.001), representing an approximate 40% reduction in depressive symptom severity. In contrast, the control group showed no meaningful change over the same period (6.8 ± 1.4 to 6.5 ± 1.5, *p* = 0.27). As shown in Figure 4, the post-intervention GDS-15 scores in the receptive music therapy group declined significantly compared with the control group, clearly demonstrating the mood-enhancing effect of the intervention. Between-group analysis using ANCOVA confirmed that the post-intervention difference in GDS-15 scores remained statistically significant after adjusting for baseline values (*F* = 10.46, *p* = 0.002). Moreover, correlation analysis revealed a positive association between improvement in mood and cognitive performance, with a Pearson correlation coefficient of *r* = 0.42 (*p* < 0.05), suggesting that participants who experienced greater reductions in depressive symptoms also demonstrated stronger cognitive gains.

At baseline, 59% of participants in the intervention group and 57% in the control group had GDS-15 scores ≥6, indicating clinically relevant depressive symptoms. The observed post-intervention reduction therefore reflects improvement primarily among participants with baseline depressive symptomatology rather than a floor effect in non-depressed individuals.

Overall, these results indicate that receptive music therapy exerts a dual therapeutic effect—alleviating depressive mood while simultaneously enhancing cognitive function. Participants frequently reported that the music sessions promoted calmness, emotional comfort, and improved sleep, which may have contributed to the observed psychological and cognitive benefits. Together, the findings reinforce the value of incorporating receptive music therapy into routine nursing care for elderly patients with T2DM and cognitive impairment as a safe, low-cost, and effective approach to supporting mental and emotional well-being.

### 3.5 Glycemic control

To determine whether receptive music therapy had any secondary influence on metabolic regulation, fasting blood glucose and HbA1c levels were evaluated at baseline and again after the eight-week intervention period. Both groups exhibited slight downward trends in glycemic parameters, although none reached statistical significance. In the intervention group, mean HbA1c declined from 7.8 ± 0.9% to 7.5 ± 0.8% (*p* = 0.11), while fasting glucose decreased from 8.4 ± 1.2 mmol/L to 8.1 ± 1.0 mmol/L (*p* = 0.14). Significant changes were not found in the control group (HbA1c = 7.8 ± 0.9% → 7.7 ± 1.0%, *p* = 0.36; fasting glucose = 8.5 ± 1.1 mmol/L → 8.4 ± 1.1 mmol/L, *p* = 0.42).

Between-group comparisons after adjusting for baseline values revealed no significant differences in either HbA1c or fasting glucose (*p* > 0.05 for all parameters). These results, summarized in Table 2, confirm that receptive music therapy did not adversely affect metabolic control or interfere with diabetes management. No adverse events, including hypoglycemia, dizziness, or fatigue, were reported throughout the study. Overall, glycemic stability was maintained across both groups, suggesting that the psychological and cognitive benefits of the intervention were achieved without compromising metabolic safety.

**Table 2:** Changes in glycemic parameters before and after intervention.

Parameter	Control (*n* = 37)	Intervention (*n* = 38)	Between-group *p*-value
HbA1c (%) – Baseline	7.8 ± 0.9	7.8 ± 0.9	0.91
HbA1c (%) – Week 8	7.7 ± 1.0	7.5 ± 0.8	0.38
Fasting glucose (mmol/L) – Baseline	8.5 ± 1.1	8.4 ± 1.2	0.82
Fasting glucose (mmol/L) – Week 8	8.4 ± 1.1	8.1 ± 1.0	0.46

Values are expressed as mean ± standard deviation (SD). No significant within-group or between-group differences were detected (*p* > 0.05 for all comparisons).

### 3.6 Participant satisfaction

Participant feedback indicated a high level of acceptance and enjoyment of the receptive music therapy sessions. More than 90% of individuals in the intervention group described the program as *pleasant* or *very relaxing*, and the majority expressed willingness to continue listening to therapeutic music beyond the study period. Subjective reports highlighted several perceived benefits, including improved sleep quality, greater emotional stability, and enhanced concentration during daily activities.

Session attendance and compliance rates were excellent, with overall adherence exceeding 95%. No participants discontinued the intervention because of discomfort, fatigue, or loss of interest. Nursing records and follow-up interviews confirmed consistent engagement throughout the eight-week program. Participants frequently commented that the structured listening environment created a sense of calmness and routine that complemented their medical and nursing care. These results demonstrate that receptive music therapy is not only clinically effective but also highly feasible and well-tolerated among elderly patients with T2DM and cognitive impairment. The strong adherence and positive satisfaction outcomes support the practical integration of music therapy into regular nursing protocols for geriatric diabetes management.

## 4 Discussion

The present randomized controlled trial demonstrated that RMT combined with usual nursing care significantly improved cognitive performance, QoL, and mood among elderly patients with T2DM and cognitive impairment. Participants receiving RMT exhibited substantial gains in global cognition, as reflected by increased MoCA scores, together with meaningful enhancements in statistically significant improvements in psychological and social domains. While no established minimal clinically important difference exists for the WHOQOL-BREF, the observed between-group differences and moderate effect sizes suggest meaningful improvements in subjective well-being without overstating clinical impact. Importantly, these benefits were achieved without adverse events or deterioration of metabolic parameters, underscoring the intervention’s safety, feasibility, and high acceptability in geriatric diabetic care.

The improvement in cognitive performance observed in this study aligns with accumulating evidence that structured music interventions can enhance neurocognitive functioning in older adults with mild cognitive impairment or dementia (12, 14, 21). Meta-analytic data indicate moderate effect sizes for music therapy on global cognition and executive processes, suggesting robust and reproducible effects across populations (13). Our finding that RMT improved MoCA scores by approximately four points within 8 weeks is consistent with prior reports showing significant improvements in attention, working memory, and visuospatial performance following similar interventions (22). The mechanisms underlying these effects may involve enhanced neural synchronization and increased cerebral perfusion within frontotemporal and limbic networks, as music listening recruits widespread cortical areas linked to attention and memory consolidation (23). Functional MRI studies have confirmed that listening to emotionally salient music increases activation in the hippocampus, prefrontal cortex, and anterior cingulate—regions particularly vulnerable to diabetic neurodegeneration (24). Thus, repetitive exposure to structured, emotionally resonant music may help restore neural efficiency through neuroplastic adaptation and dopaminergic modulation.

Parallel improvements in QoL further reinforce the holistic potential of RMT in chronic disease management. Psychological and social domain gains observed in the intervention group indicate that music therapy supports emotional regulation and interpersonal engagement—critical dimensions of well-being often diminished in T2DM (25). Previous studies have shown that music listening promotes relaxation, reduces anxiety, and enhances social connectedness through oxytocin and endorphin release (26, 27). In older adults with cognitive impairment, musical experiences evoke autobiographical memories and facilitate positive affect, thereby improving perceived life satisfaction (28). Our findings extend these benefits to the diabetic population, suggesting that RMT can mitigate diabetes-related distress and reinforce coping mechanisms. The structured, calm listening environment may provide a predictable and comforting routine that complements medical and nursing care, fostering both emotional balance and self-efficacy.

The pronounced reduction in depressive symptoms provides further support for the psychophysiological efficacy of RMT. In the present study, GDS-15 scores decreased by approximately 40% in the intervention group, indicating clinically meaningful mood improvement. This outcome parallels earlier evidence demonstrating that music therapy modulates the hypothalamic–pituitary–adrenal axis and reduces circulating cortisol, thereby alleviating stress-induced depressive symptoms (29). Neurochemical studies show that listening to pleasant music increases dopamine and serotonin availability while decreasing cortisol and noradrenaline levels, collectively fostering a neurochemical profile conducive to emotional recovery (20). The positive correlation between mood improvement and cognitive gain observed in our sample (*r* = 0.42) supports the notion that enhanced affective states contribute to better cognitive performance, likely through reduced psychological burden and improved attentional engagement.

Interestingly, RMT exerted no measurable effect on glycaemic indices such as fasting glucose or HbA1c, consistent with prior literature indicating that short-term psychosocial interventions seldom produce direct metabolic changes (30). Nonetheless, maintaining stable glycaemic control while achieving significant psychological benefits demonstrates that RMT can be safely integrated into diabetic management without interfering with pharmacological treatment or lifestyle guidelines. Previous studies in cardiac and oncology patients have similarly reported that music therapy improves mood and QoL without compromising physiological stability (31, 32). The absence of adverse events and high adherence (> 95%) observed here indicate strong feasibility for implementation in hospital wards and community nursing programs.

While receptive music therapy is often described as a safe and low-cost intervention, these assumptions warrant careful qualification. Existing literature has documented that music listening can elicit negative emotional responses, agitation, or distress in some individuals, particularly in dementia care, when music is poorly selected or insufficiently monitored. Therefore, the safety of receptive music therapy should be understood as conditional upon structured delivery, appropriate music selection, and ongoing clinical supervision. Although receptive music therapy is sometimes characterized as a low-cost intervention, the present program should be recognized as relatively intensive. Individual delivery five times per week requires substantial staff involvement and time commitment, which may limit feasibility in routine clinical settings. Therefore, the description of RMT as “low-cost” should be interpreted in relation to the absence of specialized equipment rather than overall implementation cost. Alternative delivery models warrant consideration. Group-based receptive music therapy sessions may reduce staffing demands and facilitate social engagement, though group formats may alter attentional focus and individual emotional responsiveness. Similarly, lower-frequency interventions, such as one or two sessions per week, may offer a more pragmatic balance between feasibility and benefit, but their effectiveness relative to higher-frequency programs remains uncertain. There may also be scope for hybrid or partially self-directed models, in which therapist-led sessions are combined with guided home-based listening supported by structured playlists and periodic professional monitoring. Such approaches could reduce staff burden while maintaining therapeutic structure; however, their safety, adherence, and efficacy require formal evaluation.

Future research should directly compare delivery formats and dosing strategies to inform scalable implementation. With respect to implementation, receptive music therapy may appear relatively easy to integrate into routine nursing care; however, this feasibility is contingent upon adherence to key therapeutic components. These include structured session timing, appropriate acoustic parameters, culturally familiar and emotionally neutral music selection, and delivery within a supportive clinical environment by trained staff. Consequently, while RMT is low-cost and does not require specialized equipment, it should be distinguished from informal or unsupervised music exposure when considering translation into practice. Nurses play a critical role in delivering patient-centered care and in supporting the integration of adjunctive interventions into routine clinical practice. In the present study, receptive music therapy sessions were delivered by a certified music therapist with nursing support. While it is possible that nurses could be trained to facilitate structured receptive music interventions in future practice, this was not evaluated in the current study and should be explored in dedicated implementation research. The intervention aligns with holistic nursing frameworks emphasizing psychosocial well-being, stress reduction, and individualized engagement. Furthermore, regular RMT sessions may enhance the nurse–patient relationship, increase adherence to medical regimens, and promote patient satisfactional of which contribute to improved long-term outcomes in chronic disease management.

From a mechanistic perspective, the beneficial effects of RMT may be interpreted through neuroendocrine and vascular pathways commonly disrupted in diabetes. Chronic hyperglycemia and insulin resistance impair cerebral glucose metabolism, leading to oxidative stress and microvascular injury that compromise neuronal function (3). Music-induced relaxation likely mitigates these effects by reducing sympathetic nervous activation, lowering systemic cortisol and catecholamine levels, and improving cerebral oxygenation (33). Moreover, music listening stimulates the dopaminergic reward circuitry, which modulates attention, learning, and emotional valence—core domains affected in diabetic cognitive impairment (34). Through these complementary neural and endocrine pathways, RMT provides both restorative and preventive benefits against cognitive decline.

The present findings also highlight the relevance of personalized, culturally sensitive music selection. Participants who listened to familiar or emotionally meaningful music reported stronger relaxation and engagement, consistent with previous reports that culturally congruent music evokes deeper limbic activation and greater therapeutic impact (35, 36). This observation supports the integration of individualized music libraries within nursing protocols to maximize effectiveness.

Several limitations warrant consideration. First, the sample size, though adequate for statistical power, was relatively small and the study was performed at a single center, limiting generalizability. Second, the follow-up duration was short; longer interventions are needed to assess sustained cognitive and emotional benefits. Third, although participant blinding was not feasible because of the nature of the intervention, outcome assessors and data analysts were blinded to group allocation. Nevertheless, the lack of participant blinding may have introduced a degree of performance bias, which should be considered when interpreting the findings. Future multicenter studies should incorporate larger samples, extended follow-up periods, and neuroimaging or biomarker analyses to elucidate mechanistic pathways. Moreover, comparisons between receptive and active (performative) music therapy could clarify differential therapeutic effects.

Despite these limitations, the trial provides robust preliminary evidence that receptive music therapy is a feasible, safe, and effective adjunct to nursing care in elderly patients with T2DM and cognitive impairment. The intervention improved cognitive performance, alleviated depressive symptoms, and enhanced perceived QoL without compromising metabolic stability. These findings underscore the need for holistic, integrative approaches within geriatric nursing practice that address both the physiological and psychological dimensions of chronic illness.

In conclusion, receptive music therapy offers a promising, evidence-based complement to standard diabetic care. By engaging cognitive, emotional, and neuroendocrine systems, RMT contributes to cognitive preservation, emotional well-being, and improved QoL among elderly patients with T2DM. Future large-scale studies are warranted to confirm these findings and to develop structured implementation frameworks for routine clinical use in nursing and community health settings.

## 5 Conclusion

This randomized controlled trial provides compelling evidence that receptive music therapy, when integrated with usual nursing care, significantly enhances cognitive function, QoL, and emotional well-being in elderly patients with T2DM and cognitive impairment. Over an eight-week intervention period, participants who engaged in structured, passive music listening demonstrated marked improvements in global cognition and psychological health, accompanied by a substantial reduction in depressive symptoms, while maintaining stable glycemic control. Taken together, the findings suggest that receptive music therapy may serve as a feasible adjunct to standard nursing care when delivered within a structured and supervised framework. Its clinical value lies in its potential to support cognitive and emotional well-being, rather than in assumptions of universal safety, cost-effectiveness, or scalability. From a nursing perspective, the incorporation of RMT into geriatric care routines offers a practical, holistic approach that addresses both the cognitive and psychological dimensions of chronic illness. By promoting relaxation, emotional stability, and patient engagement, RMT enhances not only individual well-being but also adherence to treatment and overall satisfaction with care. The strong feasibility and high acceptance observed in this study underscore its potential for integration into hospital wards, community health centers, and long-term care settings. Future research should extend these findings through larger, multicenter trials with longer follow-up durations and neurobiological assessments to elucidate the mechanisms linking music, mood regulation, and cognitive resilience in diabetes-related cognitive decline. Collectively, the results of this study reinforce the importance of adopting multidimensional, person-centered strategies such as RMT in geriatric nursing practice to foster mental vitality, independence, and QoL among older adults living with diabetes.

## Data Availability

The original contributions presented in the study are included in the article/supplementary material, further inquiries can be directed to the corresponding author.
